# Garretson’s Operation for the Removal of the Coccyx without Disturbance of the Perineum

**Published:** 1883-01

**Authors:** S. Parker Cottrell


Article III.

### GARRETSON'S OPERATION FOR THE REMOVAL OF THE COCCYX WITHOUT DISTURBANCE OF THE PERINEUM.

BY S. PARKER COTTRELL, M. D.

The operation devised and practiced by Dr. Garretson, of Philadelphia, for the removal of the coccyx is one of such singular simplicity and apparently so absolutely free from danger of any kind, that it needs alone to be heard of to command universal adoption; this conclusion being the opinion of all who have seen the performance.

The instrument employed for the ablation of the bone is a circular bur revolved to the extent of ten thousand revolutions to the minute by means of the surgical engine. The patient being profoundly etherized and placed partly upon the abdomen, the index finger of the left hand is oiled and passed into the rectum, thus underlying the coccyx and supporting it. Next, a bistoury is sunk through the external soft parts, an object being to strike the bone midway between its apex and base. From this the cut is extended upward and downward, with the intention of exposing the length of the part to be removed. A succeeding step consists of the introduction of retractors, by means of which the soft parts are pulled away laterally, which retraction, aided by a few touches of the knife, exposes the periosteum. Here a chisel-shaped blade is used, the periosteum being incised along its middle line, and each leaf worked over to its own side; or, if preferred, this periosteal tissue may be scraped away with a raspatory. The bone thus exposed, the operator, still having the finger in the rectum, applies the cut face of the bur at a time when the instrument is in rapid rotation. If the coccyx be deeply situated, the sense of touch, as related between the rectal finger and the hand grasping the instrument, is now depended on exclusively as to direction. If, on the contrary, the bone is but lightly covered, the surgeon may be guided wholly by sense of sight. Five minutes, six or eight at most, suffices for the bur to convert the bone into dust, taking it away completely from the deep layer of its periosteal envelope, which layer it leaves undisturbed, expressing here the great features of the operation, which features are, first, non-interference with the attachments of the posterior perineum, and, second, a wound that is strictly of a superficial expression and meaning.

The powdered bone is carefully washed out of the cavity and the parts closed, with a view of securing union by first intention, a few threads, or a delicate tube, as happens to be most convenient, being placed in that extremity of the wound which adjoins the sacrum.

Doing so delicate an operation as this just described, depending alone on the sense of touch for the management of an instrument that is revolving ten thousand revolutions a minute, may not strike the reader unfamiliar with such character of instrumentation as so easy and simple a matter, but the doubt vanishes when it is understood that a bur working with such rapidity is not to be distinguished from another strictly at rest. The writer has seen Prof. Garretson do the operation twice. In the first case, the hand-piece was held precisely as one manipulates a pen in its holder when in the act of writing; in the second instance, the bone being situated at a depth of over two inches, the hand-piece was grasped firmly in the palm. Certainly no idea as to the probability of the instrument getting away, or doing ought but the will of the surgeon, entered the mind of any of the on-lookers at the operations referred to. The undisturbed under layer of periosteum, as it is seen and felt, lying in the depth of the wound, is a matter to excite enthusiasm on the part of a professional beholder. It is next to impossible to associate the idea of danger with what is known to have been done.

Through the kindness of the family physician in attendance on the case having the deep coccyx, I am enabled to append report of progress for the first fourteen days.

Second day after operation: Patient without evidence of shock or derangement of any kind.

Third day: Considerable fever; pulse 120. Gluteal region of left side somewhat indurated. Patient restless.

Fourth day: Free suppuration at seat of tent. Pus watery, rather than laudable. Induration markedly lessened. Pulse, 97. Patient fairly comfortable; insists on being lifted to the commode.

Fifth day: Free suppuration. Slight chill.

Sixth day: Slight chill at same hour of morning. Free suppuration. Pulse, 90. Castor oil given at 10 P. M. Operated at 1 A. M. Relieved the patient of flatulency and of slight abdominal tenderness, of which she complained.

Seventh, eighth and ninth days: Discharge continues. Slight creeps each day at 11 o'clock. Pulse varying from 72 to 65. Tongue unduly dry. Administered dilute hydrochloric acid in doses of fifteen drops each three hours, and quinæ sulphas, in doses of one grain at the same intervening periods.

Tenth day: No creep. Suppuration decreasing. Wound curing most satisfactorily.

Eleventh day: Patient without discomfort of any kind. Pulse, 72. Skin cool. Tongue moist. Sat up for half an hour.

Eleventh to fourteenth day: Progress as to be expected.

---

ARTICLE IV.

